# Emergent complexity in slowly driven stochastic processes

**Published:** 2023-01-23

**Authors:** Antonio Carlos Costa, Massimo Vergassola

**Affiliations:** Laboratoire de Physique de l’Ecole normale supérieure, ENS, Université PSL, CNRS, Sorbonne Université, Université de Paris, F-75005 Paris, France

## Abstract

We consider the distribution of first passage time events in the presence of non-ergodic modes that drive otherwise ergodic dynamics on a potential landscape. We find that in the limit of slow and large enough fluctuations the distribution of first passage time events, *f*(*t*), exhibits heavy tails dominated by a power law with exponent *f*(*t*) ~ *t*^−2^, and corrections that depend on the strength and the nature of fluctuations. We support our theoretical findings through direct numerical simulations in illustrative examples.

## Introduction.—

Complex dynamics are ubiquitous in the natural world. Despite their intrinsic irregularity and unpredictability, they can nonetheless exhibit coherent and universal emergent properties. Of particular importance in the study of complex systems is the understanding of the time it takes for rare events to occur [1–3]. Notable examples include natural disasters [4] or the spreading of a virus [5]. In fact, first passage times are central to many fields within physics and beyond, with important examples stemming from chemistry, biology and finance (see, e.g., [6–12] and references therein). Biology in particular is ripe with examples where time is of the essence [13], such as fertilization [14], intracellular events [15–21], search processes [22–24], neural activity [25, 26] or population dynamics [27].

We here consider the estimation of first passage time distributions (FPTDs) from finite-time observations in an experimental context. In particular, we are interested in systems with intrinsic time scales comparable to the observation time, for which weak ergodicity breaking becomes evident [28, 29]. Such dynamics can be found for instance in glassy systems [30–33], where the time scales of equilibration are so long that one can decompose the dynamics into a stationary component and an “aging” component that breaks time-translation invariance.

Our main inspiration comes from the less traditional branch of the physics of animal behavior [34, 35]. Remarkably, recent advances in machine vision (see, e.g., [36–39]) have resulted in an explosion of high spatiotemporal resolution behavioral data. Analysis of fine-scale posture movements shows that much like the runand-tumble behavior of bacteria [40], more complex organisms also exhibit stereotyped behaviors, albeit with a more intricate structure [41–48]. The notion of stereotypy in behavior inherently stems from the time scale separation between variations on what is defined as a behavioral state, and the transitions between behavioral states, much like a particle hopping between wells in a potential landscape. For example, while foraging for food the nematode worm *C*. *elegans* transitions between coarsegrained “runs” and “pirouettes”, which are stereotyped sequences of finer scale movements [48, 49]. However, unlike the particle hopping among potential wells which has a characteristic exponential distribution of transition times, the time spent in a given behavior can be heavy-tailed (see, e.g. Fig. 4E of [48] or Fig. 3 of [50]). We here hypothesize that such heavy-tailed distributions reflect the slow continuous modulation of behavior on longer time scales, resulting from environmental factors or fluctuating internal states driven by neuromodulation, such as hunger, stress or arousal (see, e.g., [51–53]). Indeed, it has been shown that *C*. *elegans* continuously modulates its rate of reorientation events to explore larger and larger arenas in search of food [54]. In order to truly capture the multiscale nature of behavior, we therefore need to account for the fact that it can be modulated on time scales comparable to the observation time.

In this Letter, we introduce a general model of behavior in which the pose dynamics evolves in potential landscapes that fluctuate over time. We then study how these dynamics impact the estimation of the distribution of times spent in a given behavior. In the first section, we introduce our phenomenological description of the behavioral dynamics, decomposing it into ergodic dynamics on a potential landscape and the non-ergodic modulation of the landscape. We then derive a general result for the distribution of first passage times, and illustrate it through direct numerical simulations in three example systems.

## Slowly driven ergodic dynamics.—

Given a set of observations of animal locomotion (e.g. from video imaging), we consider that the dynamics can be decomposed into ergodic and non-ergodic components. The former are the state-space variables that mix sufficiently well and define the potential wells that correspond to the stereotyped behaviors; the latter non-ergodic components evolve on time scales comparable to the observation time and slowly modulate the potential landscape. The full dynamics is thus given by

(1) $$\{\begin{array}{l} \dot{\vec{X}}=F(\vec{X},\vec{\lambda })\\ \tau_{\lambda }\dot{\vec{\lambda }}=G(\vec{X},\vec{\lambda }) \end{array},$$

where $\vec{X}\in {\mathbb{R}}^{D}$ represents the ergodic components, $\vec{\lambda }\in$${\mathbb{R}}^{D_{\lambda }}$ represents the non-ergodic degrees of freedoms, *F* and *G* are nonlinear, possibly noisy, functions, and *τ*_*λ*_ is assumed to be of the order of the measurement time *T*_exp_, *τ*_*λ*_ = 𝒪(*T*_exp_), such that the $\vec{\lambda }$ dynamics do not mix. Given the time scale separation between the dynamics of $\vec{X}$ and $\vec{\lambda }$, we assume that the dynamics of $\vec{X}$ is well approximated by quasi-stationary Fokker-Planck dynamics $\dot{\rho }=ℐ\rho$, where ℐ represents the Fokker-Planck operator. Since we are primarily interested in the long time scale behavior of the system, we consider a projection of the dynamics onto the slowest mode of ℐ, yielding a generalized Langevin equation [55, 56] with history-dependent friction and fluctuations. Assuming that we can sample the system on a time scale longer than the noise correlation time, we obtain an effective overdamped description:

(2) $$\dot{\vec{X}}=F(\vec{X},\vec{\lambda })\Rightarrow \dot{x}=-\partial_{x}U(x,\lambda )+\sqrt{2T_{x}}\eta_{x}(t),$$

where *T*_*x*_ captures the effective temperature, *η*_*x*_ is Gaussian white noise, and *λ* is a slow control parameter that modulates the effective potential landscape on slow time scales. Similarly, we consider that *λ* also obeys an effective overdamped Langevin equation,

(3) $$\dot{\lambda }=-\tau_{\lambda }^{-1}\partial_{\lambda }V(\lambda )+\sqrt{2T_{\lambda }\tau_{\lambda }^{-1}}\eta_{\lambda }(t)$$

where *V* is assumed to be uncoupled from the dynamics of *x* for simplicity, *T*_*λ*_ captures the degree of fluctuations in *λ* and *η*_*λ*_ is Gaussian white noise.

## First passage time distributions.—

We are primarily interested in studying the time spent in a given behavioral state. Within the context of the Langevin dynamics of Eq. 2, this is given by the first passage time to reach an energy barrier *x*_*f*_ from the bottom of the potential *x*_0_, defined as,

(4) $$\tau_{x_{0},x_{f}}(\lambda )=\text{inf}\left\{\tau :x(t+\tau ,\lambda )=x_{f}|x(t,\lambda )=x_{0}\right\}.$$

Despite the general interest in this concept, finding analytical expressions for the density of first passage time events is generally a formidable task [57]. Remarkably few closed-form expressions for the FPTD are known, with most results concerning only the mean first passage time (MFPT) which is more tractable (see, e.g., [1, 6, 9]). However, the MFPT provides only limited information, especially when multiple time scales are involved [15]. Here, we are interested in studying the behavior of the full first passage time distribution, with particular focus on its long time behavior in the presence of weakly non-ergodic dynamics, Eqs. 2 and 3.

As previously discussed, the measurement time *T*_exp_ essentially separates ergodic from non-ergodic dynamics. In addition, it also sets a lower bound on the slowest observed hopping rates $\omega_{\text{min}}\sim T_{\text{exp}}^{-1}$, such that when *τ*_*λ*_ = 𝒪(*T*_exp_) we can make an adiabatic approximation and assume that transition events occur within a nearly static potential. For a given hopping rate *ω*, the first passage time distribution is given by

$$f(t,\omega )=\omega e^{-\omega t},$$

where $\omega \left(\lambda \right)=1/\tau_{x_{0},x_{f}}\left(\lambda \right)$ is the dominating slow kinetic transition rate which implicitly depends on the dynamics of *λ*. When we allow *λ* to fluctuate slowly, the distribution of first passage times *f*(*t*) is given by the expectation value of *f*(*t*, *ω*) over the distribution of *ω*, *p*(*ω*), weighted by the effective number of transition observed within *T*_exp_, which is proportional to *ω*. Marginalizing over *ω* we get

(5) $$f(t)\sim \int_{\omega_{\text{min}}}^{\omega_{\text{max}}}p(\omega )\omega^{2}e^{-\omega t}d\omega .$$

While the barrier height is going to depend on the dynamics of a slow control parameter *λ*, the tail of the distribution is going to be dominated by instances in which the barrier height is the largest, motivating the use of Kramers approximation (see, e.g., [2]),

(6) $$\omega (\lambda )=\omega_{0}\text{exp}\left\{-\frac{\Delta U(\lambda )}{T_{x}}\right\},$$

where Δ*U*(*λ*) = *U*(*x*_*f*_, *λ*)−*U*(*x*_0_, *λ*) and *ω*_0_ is a constant. For multiple realization of Eq. 3 with different initial conditions, the distribution of *λ* is given by the Boltzmann weight [58],

(7) $$p(\lambda )\sim \text{exp}\left\{-\frac{V(\lambda )}{2T_{\lambda }}\right\}.$$

Leveraging Eqs.S1,6,7 we can obtain an asymptotic approximation of the FPTD in the large *t* limit (see Supplemental Material),

(8) $$f(t)\sim t^{-2}\text{exp}\left\{-\frac{V\left(\Delta U^{-1}\left(T_{x}\text{log}\left(\omega_{0}t\right)\right)\right)}{2T_{\lambda }}\right\},$$

where Δ*U*^−1^(·) represents the inverse function of the potential difference defined by Eq. 6 and we have kept only the dominant order of the asymptotic approximation (see Supplemental Material). For very general conditions on *V* (*λ*) and *U*(*x*, *λ*), we thus get *f*(*t*) ~ *t*^−2^ for *t* → ∞ and *T*_*λ*_ ≫ 1. In the following section we will demonstrate the validity of this result in three illustrative examples.

### Illustrative examples: Slowly-driven harmonic oscillator.—

**FIG. 1.**
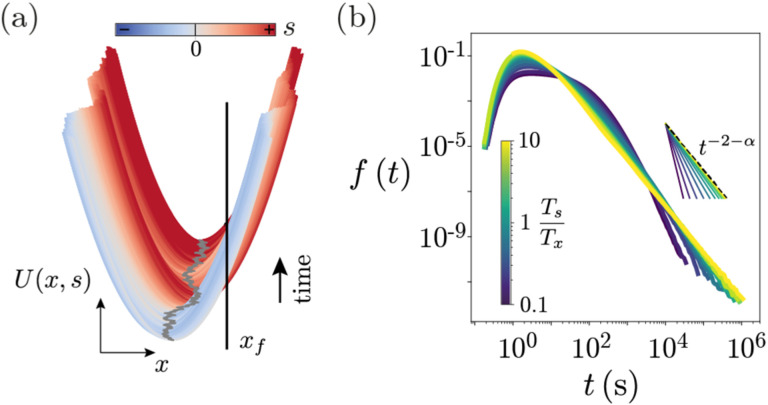
Heavy-tailed first passage time distributions for a slowly-driven overdamped harmonic oscillator. (a) We simulate the dynamics of a particle in a harmonic oscillator while slowly driving the potential landscape, and estimate the distribution of times it takes to reach *x*_*f*_. The gray line represents the minimum of potential, *x*_0_ = *s*, and the color scheme different values of *s*. (b) FPTDs obtained from direct numerical simulations of Eq. 9 for different values of the temperature *T*_*s*_ that controls the level of fluctuations for the parameter driving the slow variations of the potential landscape. As predicted, the tail of the distribution behaves as a power law with an exponent *f*(*t*) ~ *t*^−2−*α*^, with $\alpha =\frac{T_{x}}{2T_{s}}$. The color scheme represents different ratios of temperatures, and the black dashed line the *T*_*s*_ →∞ limit.

Consider that *x* evolves in a harmonic potential, *U*(*x*, *s*) = (*x* − *sx*_*f*_)^2^, that is driven by a slow parameter *s* that fluctuates within *V* (*s*) = *s*^2^*/*2, pushing *U*(*x*, *s*) closer or further from *x*_*f*_ in a time scale *τ*_*s*_, Fig. 1(a). The equations of motion are given by a set of Ito stochastic differential equation, corresponding to coupled Ornstein-Uhlenbeck processes,

(9) $$\{\begin{array}{l} dx_{t}=-\left(x_{t}-s_{t}x_{f}\right)dt+\sqrt{2T_{x}}dW_{t}\\ ds_{t}=-\tau_{s}^{-1}s_{t}+\sqrt{2T_{s}\tau_{s}^{-1}}dW_{t} \end{array},$$

where *T*_*x*_ and *T*_*s*_ captures the degree of fluctuations, *dW*_*t*_ is a Wiener Gaussian white noise process. We are interested in the density of first passage time events from the minimum of the potential *x*_0_ = *s* to *x*_*f*_ = 1, for which it is challenging to find a closed form analytical expression, even when s(t)=s∈ℝ [57]. In the Supplemental Material, we derive the FPTD in Laplace space [59] and leverage it to estimate the FPTD through numerical inversion [60] for varying values of *τ*_*s*_ (as in Ref. [61]), see Fig. S2. We find that when *s* fluctuates fast enough, *τ*_*s*_ → 0, we can average out *s* and get the simpler dynamics $dx_{t}=-\left(x_{t}-\langle s\rangle x_{f}\right)dt+\sqrt{2T_{x}}dW_{t}$. In this case, the FPTD is well approximated by *f*(*t*) ≈ *f*(*t*, 〈*ω*〉) = 〈*ω*〉*e*^−〈*ω*〉i*t*^, where 〈*ω*〉 is the average hopping rate which is set by 〈*s*〉. Even when *τ*_*s*_ > 0 but short, it is possible to obtain a self-consistent Markovian dynamics for *x*(*t*) (see e.g., [1]). In this case, the distribution of first passage times is still dominantly exponential, but with a corrected first passage time which depends on the ratio of temperatures *T*_*s*_*/T*_*x*_ and the slow time scale *τ*_*s*_. However, as we have shown in the previous section *τ*_*s*_ is large enough, *τ*_*s*_ ~ *T*_exp_, the distribution of first passage times becomes heavy-tailed. In this limit, we can leverage Eq. 8 to derive an asymptotic approximation to the distribution of first passage times. The tail of the distribution will be dominated by low *ω* values, which correspond to |*s*| ≫ 1. In this limit, the barrier height primarily behaves as Δ*U*(*s*) = *s*^2^*/*2 + 𝒪(*s*). In addition, since *V* (*s*) = *s*^2^*/*2, we see that *V* (Δ*U*^−1^(*x*)) = *x* and Eq. 8 yields (see Supplemental Material),

(10) $$f(t)\sim t^{-2-\frac{T_{x}}{2T_{s}}},$$

which matches what we obtain from direct numerical simulations of Eq. 9, Figs. 1(b),S2,S3(a).

### Illustrative examples: Slowly-driven double-well potential.—

**FIG. 2.**
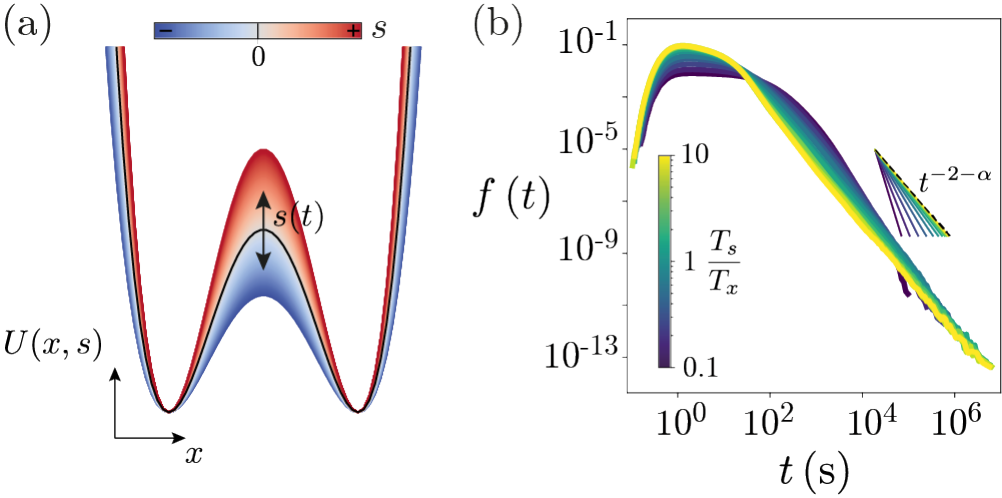
Heavy-tailed first passage time distribution of a slowly-driven double-well potential. (a) Schematic of the variation in the double-well potential with *s* (colored from blue to red; the black line represents *s* = *μ*_*s*_). (b) FPTDs from direct numerical simulations of Eq. 11 for different values of *T*_*s*_. As expected, the tail of the distribution behaves as a power law *f*(*t*) ~ *t*^−2−*α*^, where $\alpha =\frac{T_{x}}{4T_{s}}$ (colored line). The black dashed line represents the *T*_*s*_ →∞ limit.

We now consider a symmetric double-well potential in which the barrier height is slowly modulated according to an Ornstein-Uhlenbeck process, Fig. 2(a),

(11) $$\{\begin{array}{l} dx_{t}=-4s_{t}^{2}x_{t}{\left(x_{t}-1\right)}^{2}dt+\sqrt{2T_{x}}dW_{t}\\ ds_{t}=-\tau_{s}^{-1}\left(s_{t}-\mu_{s}\right)dt+\sqrt{2T_{s}\tau_{s}^{-1}}dW_{t} \end{array},$$

where all the parameters are the same as in Eq. 9 with an extra *μ*_*s*_ that represents the expectation value of *s*, which we set as *μ*_*s*_ = 1. In this case, we have a quartic potential for *x*, *U*(*x*, *s*) = *s*^2^(*x*^2^ − 1)^2^, which yields Δ*U*(*s*) = *s*^2^. Since *V* (*s*) = *s*^2^*/*2, we see that *V* (Δ*U*^−1^(*x*)) = *x/*2 and Eq. 8 yields (see Supplemental Material),

(12) $$f(t)\sim t^{-2-\frac{T_{x}}{4T_{s}}},$$

matching what we find through direct numerical simulations of Eq. 11, Figs. 2(b),S3(b)

### Illustrative examples: Slowly-driven rugged parabolic potential.—

**FIG. 3.**
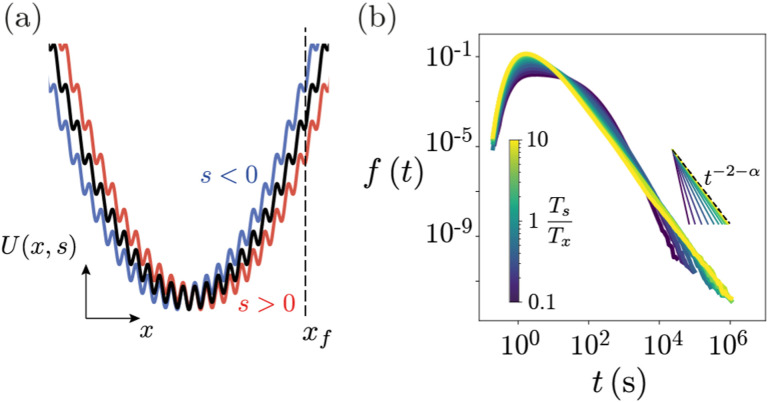
Heavy-tailed first passage time distribution in slowly driven rugged parabolic potential. (a) We estimate the first passage time to reach *x*_*f*_ from the global minimum of a rugged parabolic potential. (b) FPTDs from direct numerical simulations of Eq. 13 for different values of *T*_*s*_. As expected, the tail of the distribution behaves as a power law *f*(*t*) ~ *t*^−2−*α*^ (colored lines) with $\alpha =\frac{T_{x}}{2T_{s}}$. The black dashed line corresponds to the *T*_*s*_ →∞ limit.

Finally, we consider a rugged parabolic potential as a simple model of the rough energy landscapes found across complex systems, from glasses to proteins (see, e.g., [19, 20, 62]). We cons truct a rugged landscape by superimposing a sinusoidal perturbation onto a harmonic potential [63], *U*(*x*, *s*) = *U*_0_(*x*, *s*) + *U*_1_(*x*), where *U*_0_ (*x*, *s*) = (*x*, *s*)^2^/2 and *U*_1_(*x*) = −cos(2π*kx*)/(*kπ*). The corresponding dynamics are given by,

(13) $$\{\begin{array}{l} dx_{t}=-\left(x_{t}-s_{t}+2\;\text{sin}\left(2\pi kx_{t}\right)\right)dt+\sqrt{2T_{x}}dW_{t}\\ ds_{t}=-\tau_{s}^{-1}s_{t}+\sqrt{2T_{s}\tau_{s}^{-1}}dW_{t} \end{array},$$

where *k* sets the number of smaller barriers between the global minimum of the potential and *x*_*f*_ = 1. We set *k* = 10 resulting in a rugged potential as illustrated in Fig. 3(a). In this case, since *U*(*x*, *s*) is not as simple as before, it is more challenging to derive the correction terms to the power law. However, it has been shown [63] that by spatial averaging of *U*_1_(*x*) = −cos(2*πkx*)*/*(*kπ*) over one period, the resulting hopping rate is simply corrected by a constant prefactor $\omega =I_{0}^{-2}\left(k^{-1}\pi^{-1}T_{x}^{-1}\right)\omega_{0}$, where *I*_0_ is the modified Bessel function and *ω*_0_ is the hopping rate in the absence of the sinusoidal perturbation (from *U*_0_(*x*, *s*) = (*x* − *s*)^2^*/*2). As such, we expect the asymptotic behavior of *f*(*t*) to be the same as for the slowly driven harmonic potential, Eq. 10. Indeed, this is what we observe in Figs. 3(b),S3(c).

## Discussion.—

Inspired by quantitative analysis of animal behavior, we here examined how the existence of slow non-ergodic modes impacts the statistics collected experimentally, focusing on the distribution of first passage time events. Our results show the emergence of heavy-tailed distributions. In particular, we find that the distribution asymptotes to a power law with an exponent *f*(*t*) ~ *t*^−2^ in the limit of large fluctuations, regardless of the details of the dynamics. As remarked in the Introduction, our results have important implications to a wide variety of fields, and we here discuss some of these in detail.

In the context of animal behavior, heavy-tailed first passage times with an exponent *f*(*t*) ≈ *t*^−2^ have been found extensively across multiple species, from bacteria [64], termites [65] and rats [66] to marine animals [67, 68], humans [69] and even fossil records [70]. In the context of search behaviors (e.g., when foraging for food), such observations have led researchers to hypothesize that Lévyflights (power law distributed run lengths) are efficient search strategies and thus evolutionarily favorable [71–75]. However, we here show that such fat tails may emerge when the animal is continuously adapting its behavior (slowly modulating the potential landscape), even in the absence of external drives. We therefore predict that disrupting the internal mechanisms for slow modulation of behavior (e.g. neuromodulatory pathways) should result in distribution of first passage times that have exponential tails.

Power laws have been observed in a wide variety of systems, from solar flares [76, 77] to the brain [78] and different hypotheses have been put forward to explain their emergence (for a review see e.g. [79]). Among these, work inspired by phase transitions in statistical mechanics associates power laws to “criticality”, mostly due to the fact that models inferred from the data appear to require fine-tuning of the model parameters to a special regime between two qualitatively different “phases” (see, e.g., [80]). However, as we have shown here, power laws can emerge without fine tuning and far from “criticality”. Indeed, slow modes that evolve on time scales comparable to the observation time are challenging to infer from data, and can give rise to best-fit models that appear “critical”. While some of the arguments we have put forward have also been proposed in other contexts [22, 81–83], we here place them into the framework of out-of-equilibrium statistical mechanics, explicitly connecting the long time scale emergent behavior with the underlying effective fluctuations. In addition, unlike other approaches [82, 84], our framework does not require explicit external drives, but simply collective modes that evolve in a weakly non-ergodic fashion.

Our starting point is an effective description of the long time scale dynamics, and further work will be required to fully bridge between microscopic dynamics, and the emergent long time behavior of the first passage time distribution that we uncovered. For example, we find that for intermediate values of 1 ≪ *T*_*λ*_ ≪ *T*_exp_ the FPTD behaves as a truncated power law with an effective exponent that is slightly smaller that −2 (see Supplemental Material), which goes beyond arguments presented here. What are the minimum *τ*_*λ*_ and *T*_*λ*_ for power laws to be measurable, and how do simple exponentials (*T*_*λ*_ ≪ *T*_exp_) transition to power law behavior? These are important questions if one hopes to test our predictions in an experimental context (using for example a set-up akin to the ones used to test stochastic resonance [85, 86]). Additionally, we note that when *T*_*λ*_ ≫ *T*_exp_, the distribution of initial conditions determines the emergent behavior, see Fig. S4. Inspired by experiments in animal behavior, which are typically done with multiple animals, we here assume that the initial condition for the slow mode is sampled according to its Boltzmann distribution $\lambda (t=0)\sim e^{-\frac{V(\lambda )}{2T_{\lambda }}}$, reflecting the variability across individuals. In this case, the emergent behavior we have derived holds true from *τ*_*λ*_ ~ *T*_exp_ to *τ*_*λ*_ → ∞. However, if the variability across experiments is smaller than that of the Boltzmann distribution, the *τ*_*λ*_ → ∞ limit will differ from the behavior at *τ*_*λ*_ ~ *T*_exp_. Indeed, if the variance of the initial distribution of *λ* is smaller than that of the Boltzmann distribution, the temperature *T*_*λ*_ in our derivation should be changed to a new effective temperature $T_{\lambda }^{0}<T_{\lambda }$ reflecting the lower variance of the initial conditions. Making this transformation we still get a power law distribution of first passage times, but with a modified exponent that reflects the lower variance (see Supplemental Material).

To conclude, we have considered the effect of slow non-ergodic modulations and theoretically captured their effects on the distribution of first passage times, a result that we believe is widely relevant to a range of natural systems.

## Supplementary Material

1
