# Supplementary material for: Fluctuating landscapes and heavy tails in animal behavior
Source: ArXiv. 2024 Apr 16:arXiv:2301.01111v4. Preprint. [Version 4] (PMC9900967)
Supplement: Supplement 1 [file NIHPP2301.01111v4-supplement-1.pdf]

SUPPLEMENTAL MATERIAL

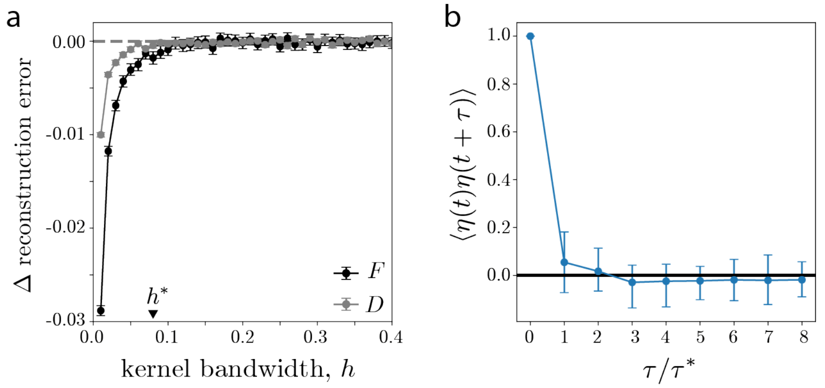

FIG. S1. **Details of the stochastic model inference in *C. elegans* wild type worms.** (a) Change in reconstruction error $\Delta\xi = \xi(h + \delta h) - \xi(h)$ of the drift F and diffusion D coefficients depending on the bandwidth of the kernel used to perform the Kramers-Moyal averages [37] (see Appendix A). For each value of bandwidth h, we simulate trajectories using the estimated drift and diffusion coefficients. We then re-infer the drift and diffusion coefficients from the simulated trajectories and compare them against the parameters obtained from the original time series to get the reconstruction error, Eq. A2 (Δ-algorithm in [37]). We chose $h^* = 0.08$ as the lowest h value when the reconstruction error stops changing (when Δ reconstruction error ≈ 0). (b) Autocorrelation function of the residuals $\eta(t)$ after fitting Eq. 2 to time series of $\phi_2(t)$. At the sampling time τ^* the noise decorrelates, thus justifying the white noise approximation. Error bars correspond to 95% confidence intervals bootstrapped over 1000 simulations of randomly sampled worms.

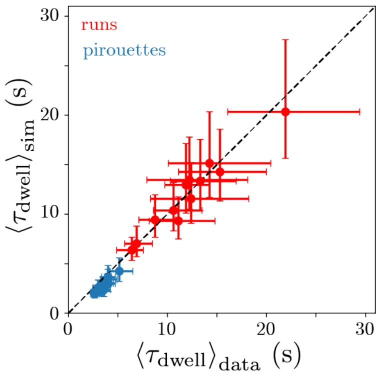

FIG. S2. **Simulations from Eq. 2 accurately predict the average time spent in a given behavioral state for *C. elegans* wild type worms**. Each point corresponds to a worm in a particular state, and the error bars correspond to 95% confidence intervals bootstrapped over events.

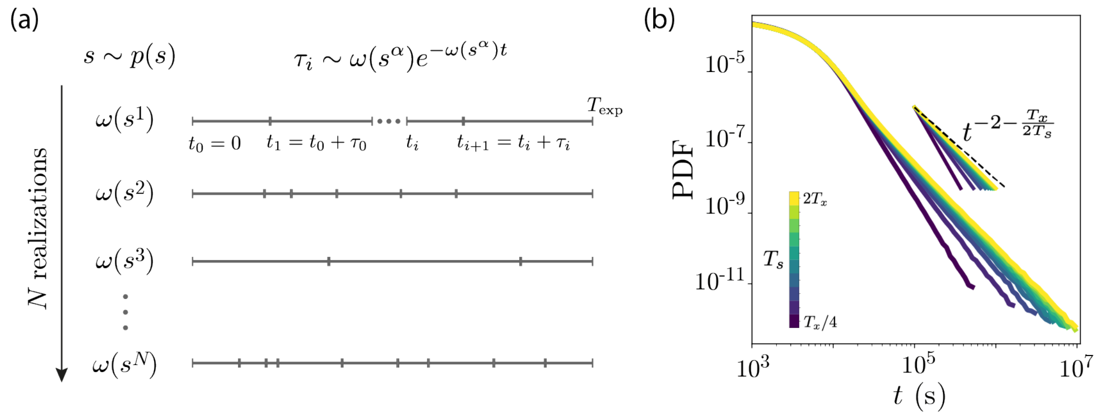

FIG. S3. **Heavy-tailed first passage time distribution in Poisson process with varying hopping rates.** (a) Schematic of the simulation process. For each realization, we sample s according to the Boltzmann distribution, $p(s)$. The hopping rate corresponding to a particular sample s^i is then determined by the backward Kolmogorov equation, Eq. B2, and event durations are sampled according to the first passage time distribution $f(t,\omega) = \omega e^{-\omega t}$ until reaching the experimental timescale $T_{\rm expt}$. This process is then repeated over N =50,000 realizations (see Appendix A). (b) Probability density function (PDF) of first passage times for the Poisson process with varying hopping rates. As predicted, we obtain a power law with an exponent $f(t) \sim t^{-2-\frac{T_x}{2T_s}}$.

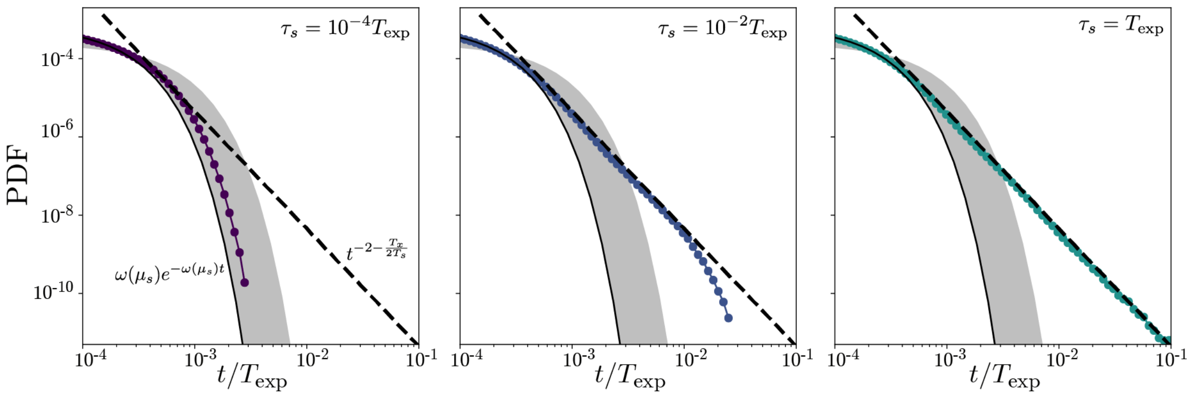

FIG. S4. **Emergence of power-law tails in the first passage time distributions for the slowly-driven double well dynamics of Eq. 12 as a function of τ_s.** For $\tau_s \ll T_{\rm expt}$ (left) the potential relaxes to its mean value faster than the hopping timescale, resulting in exponential behavior with a decay rate corresponding to the mean value $\omega(\mu_s)$. The black dashed line and gray shaded area correspond to $f(t,\omega) = \omega e^{-\omega t}$ with $\omega = \omega(\mu_s)$ and $\omega(\mu_s + \sqrt{T_s})$ respectively. As τ_s increases, the regime in which we observe power law behavior grows, and for intermediate τ_s we obtain a truncated power law with an exponential tail starting at $t \sim \tau_s$ (middle). Finally, when $\tau_s = T_{\rm expt}$ the measured tail of the distribution is power-law distributed (right). The black dashed line corresponds to our prediction $t^{-2-\frac{T_x}{2T_s}}$ and $T_s = T_x/2$.

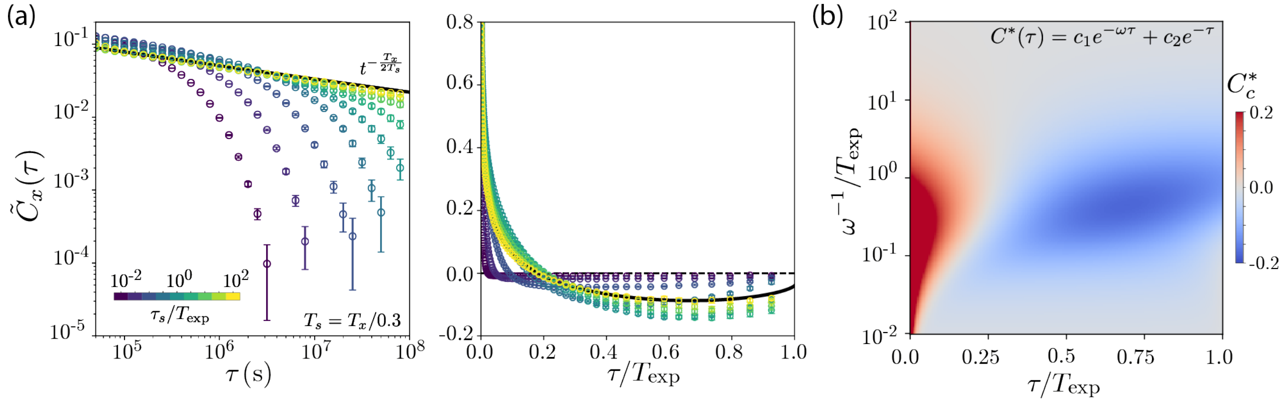

FIG. S5. **Dependence of the autocorrelation function on τ_s.** (a-left) Non-connected correlation function for $T_s = T_x/0.3$ and varying τ_s. When τ_s is small, the tail of the correlation function is exponential, with a decay rate that is given by the maximum barrier height attained at this temperature. As τ_s grows, the variation of the potential landscape becomes slower and slower and our asymptotic approximation correctly predicts the emergent power law behavior at large τ_s. (a-right) Connected correlation function for $T_s = T_x/0.3$ and varying τ_s, estimated directly from the time series data. Our adiabatic approximation correctly predicts the correction to the correlation function for large τ_s. We normalize both correlation functions by their value at $\tau = 1\,\text{lag} = 5 \times 10^{-4} T_{\rm expt}$. Notably, the correlation function exhibits finite-size corrections even when it has exponential tails, as long as the timescale of the exponential decay is comparable to the measurement time scale. Error bars represent 95% confidence intervals bootstrapped across 50,000 simulations. (b) We examine the finite-size correction to the correlation function when there is no time dependence to the hopping rate ω. For a system with a single fixed energy barrier, we would expect that the correlation function would be given by $C_x(\tau) = c_1 e^{-\omega\tau} + c_2 e^{-\tau}$ [38, 59], where $\sum_i c_i = 1$ and we take the intrinsic time scale of relaxation to a well to unity without loss of generality. As expected, even when the correlation function has exponential tails we observe the appearance of finite-size effects when $0 \ll \omega^{-1} \lesssim T_{\rm expt}$. Notably, when $\omega^{-1} \to \infty$ these finite-size effects are less apparent since only the short timescale survives. In contrast, when we allow the hopping rate to fluctuate in time we effectively generate a continuum of time scales such that, even when $T_s \to \infty$, finite-size effects are still apparent, Fig. 5(c).

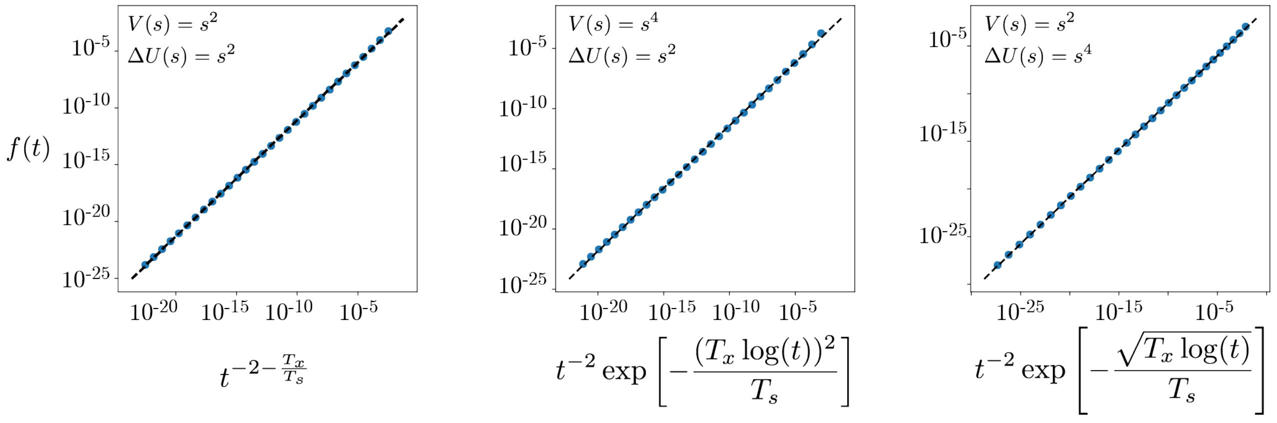

FIG. S6. Numerical integration of $f(t)$ for different choices of $V(s)$ and $\Delta U(s)$, compared to the asymptotic approximation of Eq. C5 (black dashed line) with $T_x = 0.1$ and $T_s = 0.2$. We numerically integrate Eq. C1 with $\Delta U(s) = s^k$ and $V(s) = s^n$, through a Riemann sum using the midpoint rule from $\omega_{\rm min} = 5 \times 10^{-10}$ to $\omega_{\rm max} = 1$ with $\Delta\omega = 10^{-9}$.

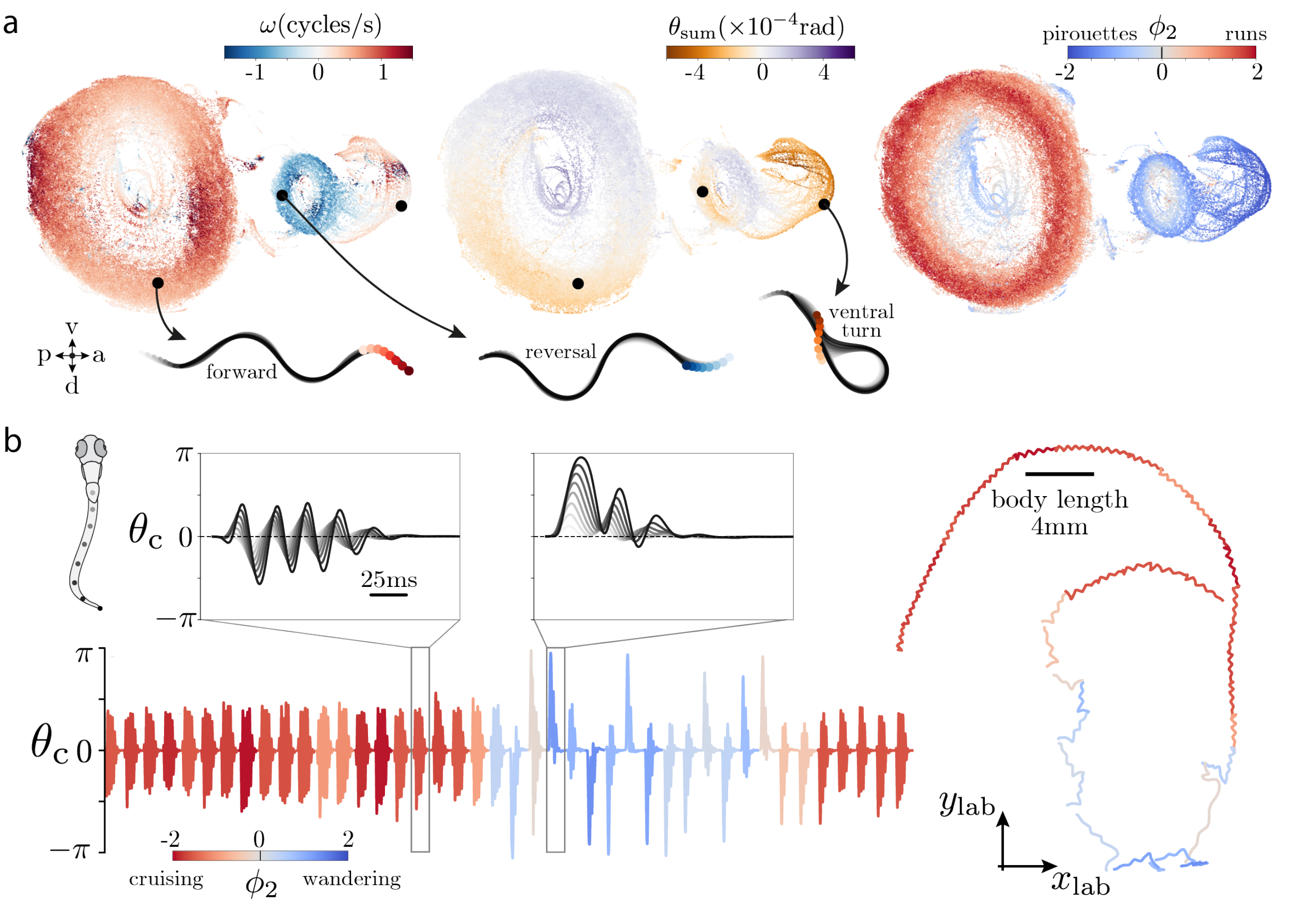

FIG. S7. **Details of the analysis of posture dynamics in *C. elegans npr-1* mutants and larval zebrafish.** (a) From videos of *npr-1* mutants crawling on a food-rich environment, we proceed as in Fig. 2, extracting the body posture (using `wormpose` [3]) and reconstructing a maximally-predictive space of posture sequences [13, 19] with $K^* = 0.4\,\mathrm{s}$ (see Appendix A). We show a 2-dimensional projection of the state-space, obtained through UMAP, and color code this space either according to the body wave phase velocity ω [33] (left), the overall body curvature (sum of the tangent angles along the body) θ_{sum} (middle), and the projection along the slowest eigenvector of the inferred transition matrix ϕ_2 (right), obtained with $\tau^* = 0.5\,\mathrm{s}$ (see Appendix A). In addition, we show a few example sequences of postures X_{K^*}, illustrative of different behaviors: forward, reversal and ventral turn (colored circle indicates the head-position, and light-to-dark color indicates the passage of time). Notably, we observe that the overall dynamics is somewhat similar to what is observed in wild type N2 worms off-food (see Fig.1 in [19]), except for the notable absence of dorsal turns. As in Fig. 2, we find that ϕ_2 captures transitions between forward "runs" and combinations of reversals (negative ω) and turns (large θ_{sum}) that constitute "pirouettes". We obtain K^*, N^* and τ^* in the same way as for the wild type dataset (see Appendix A). (b) We collected data from [64] in which larval zebrafish are exposed to a chasing dot stimulus for 5 s every 2 min for at least 1 hour. The fish move in discrete tails bursts (bouts), interrupted by periods in which the tail is immobile. A custom tracking algorithm used in [63] identifies each bout and collects the position of 9 points along the tail at 700 Hz during each bout. The posture is represented as the cumulative tail angle θ_c from head to tail, sampled for 175 frames (enough to capture the tail's relaxation). From the bout dynamics, we proceed as for *C. elegans* [19]: we identify maximally-predictive sequences of $K^* = 5$ bouts, and infer the slow dynamics ϕ_2 though the eigenspectrum of the inferred Markov chain with $\tau^* = 3$ bouts (see Appendix A). We plot an example sequence of ≈ 40 bouts, in which the cumulative tail angles are color coded by the projection along ϕ_2, as well as the resulting trajectory of the head position (right). The slow dynamics ϕ_2 corresponds to a "wandering-cruising" axis, in which the fish either engages in bout sequences with large orientation changes ("wandering"), or performs sequences of smoother forward bouts ("cruising") that result in more persistent trajectories.
