# Supplementary material for: Emergent complexity in slowly driven stochastic processes
Source: ArXiv. 2023 Jan 23:arXiv:2301.01111v2. Preprint. [Version 2] (PMC9900967)
Supplement: Supplementary file 1 [file NIHPP2301.01111V2-supplement-1.pdf]

Supplemental material for: Emergent complexity in slowly driven stochastic processes

General derivation.—We here derive the expression for the first passage time distribution (FPTD) in a fluctuating potential landscape. As discussed in the main text, we consider the adiabatic limit in which the FPTD can be approximated by,

$$f(t) \sim \int_{\omega_{\min}}^{\omega_{\max}} p(\omega)\omega^2 e^{-\omega t} d\omega. \tag{S1}$$

Since $\omega = \omega_0 \exp\{-\Delta U(\lambda)/T_x\}$, we have $\lambda(\omega) = \Delta U^{-1}(-T_x \log(\omega/\omega_0))$, where ω_0 is a typical (fast) frequency of the hopping dynamics [87]. The distribution $p(\omega)$ obeys $p(\omega)d\omega = p(\lambda)d\lambda$, and it is thus given by

$$p(\omega) \sim \exp\left\{-\frac{V(\lambda(\omega))}{2T_\lambda}\right\} \frac{T_x/\omega}{\partial_\lambda \Delta U(\lambda)}\,.$$

Plugging this into Eq. S1, we get

$$f(t) \propto \int_{\omega_{\min}}^{\omega_{\max}} \exp\left\{-\frac{V(\lambda(\omega))}{2T_\lambda}\right\} \frac{T_x}{\partial_\lambda \Delta U(\lambda)} \omega e^{-\omega t} d\omega. \tag{S2}$$

The exponential factor $e^{-\omega t}$ restricts the contributions to $\omega \sim 1/t$, which motivates the change of variable $\omega = \frac{\theta}{t}$. The above integral is then recast in the form

$$f(t) \propto t^{-2} \int_{\theta_{\min}(t)}^{\theta_{\max}(t)} \frac{\exp\left\{-\theta - \frac{V(\lambda(\theta))}{2T_\lambda} + \log(\theta)\right\}}{\partial_\lambda \Delta U(\lambda(\theta))} d\theta, \tag{S3}$$

where $\lambda(\theta) = \Delta U^{-1}\left(-T_x \log\left(\frac{\theta}{\omega_0 t}\right)\right)$, $\theta_{\min}(t) = \omega_{\min} t$ and $\theta_{\max}(t) = \omega_{\max} t$.

To grasp the structure of the integral, it is convenient to consider first the special case where V and ΔU can be written as a power series expansion $V(\lambda) \sim a\lambda^n$ and $\Delta U(\lambda) \sim b\lambda^n$, $a, b \in \mathbb{R}$ with an equal dominant (at large values of the argument, see below) exponent n. The integral reduces then to the form

$$f(t) \propto t^{-2-\frac{aT_x}{2bT_\lambda}} \int_{\theta_{\min}}^{\theta_{\max}} \frac{\theta^{1+\frac{aT_x}{2bT_\lambda}} e^{-\theta}}{\left(-\log\left(\frac{\theta}{\omega_o t}\right)\right)^{1-\frac{1}{n}}} d\theta\,.$$

It remains to verify that the time dependencies at the denominator of the integrand and the limits of integration do not spoil the behavior at large times. This is verified by noting that the numerator of the integrand has the structure of an Euler-Γ function of order $2 + \frac{aT_x}{2bT_\lambda}$. The integrand has its maximum at $\theta^* = 1 + \frac{aT_x}{2bT_\lambda}$, decays over a range of values of order unity and vanishes at the origin. In that range, the argument of the power at the denominator $\log(\omega_0 t) - \log(\theta) \simeq \log(\omega_0 t)$, which yields the final scaling with subdominant logarithmic corrections

$$f(t) \sim t^{-2-\frac{aT_x}{2bT_\lambda}} \times \log(\omega_0 t)^{\frac{1}{n}-1}. \tag{S4}$$

To complete the argument, we note that the time dependency of $\theta_{\min}$ is not an issue as long as values $\theta \sim O(1)$ are in the integration range. In practice, this means that the minimum hopping rate $\omega_{\min}$ should be comparable to (or larger than) the measurement time, $\omega_{\min}^{-1} \sim \mathcal{O}(T_{\exp})$.

Before moving to the general case, two remarks are in order. First, for $\omega_0 t \gg 1$ the functions V and $\partial_\lambda \Delta U$ that appear in Eq. S3 have their argument $\lambda \gg 1$. The dominant behavior of the two functions should then be understood for large values of their arguments. Second, the denominator $\partial_\lambda \Delta U$ could *a priori* be included in the exponential at the numerator but this does not modify our conclusion. It is indeed easy to verify that the maximum θ^* and the decay range would not be not shifted at the dominant order (and this holds also for the general case considered hereafter).

We can now consider the general case with different dominant exponents $V(\lambda) \sim a\lambda^n$ and $\Delta U(\lambda) \sim b\lambda^k$, $a, b \in \mathbb{R}$. The argument of the exponential in Eq. S3

$$L(\theta) = -\theta - \frac{V(\lambda(\theta))}{2T_\lambda} + \log(\theta)\,, \tag{S5}$$

has its maximum at θ^*, defined by the implicit equation

$$\theta^* = 1 + \frac{T_x}{2T_\lambda}\frac{\partial_\lambda V(\lambda(\theta^*))}{\partial_\lambda \Delta U(\lambda(\theta^*))} = 1 + \frac{T_x}{2T_\lambda}\frac{an}{bk}\lambda^{n-k}\,,$$

where we have used

$$\partial_\theta V(\lambda) = \partial_\lambda V(\lambda) \times \frac{d\lambda(\theta)}{d\theta}\,; \quad \frac{d\lambda(\theta)}{d\theta} = -\frac{T_x/\theta}{\partial_\lambda \Delta U(\lambda)}\,.$$

For $n < k$, the maximum $\theta^* \simeq 1$ and the integrand decays in a range of order unity. Indeed, the dominant order of the derivatives $\partial^p L$ ($p \geq 2$) at $\theta = \theta^*$ coincide with those of $\log(\theta)$. It follows that $L(\theta) - L(\theta^*) \simeq \log(\theta/\theta^*) - (\theta - \theta^*)$. The resulting integral over θ is an Euler Γ-function of order two, which indeed forms at values $O(1)$. In that range, $\lambda \sim \left(\frac{T_x}{b}\log(\omega_0 t)\right)^{1/k}$ and the integral is then approximated by $\exp\{L(\theta^*)\}$ and the function $f(t)$ in Eq. S3 by

$$f(t) \sim t^{-2}\exp\left\{-\frac{a\left(\frac{T_x}{b}\log(\omega_0 t)\right)^{n/k}}{2T_\lambda}\right\}\,.$$

The factor at the denominator in Eq. S3 is $\mathcal{O}\left[\exp\{(1/k-1)\log[\log(\omega_0 t)]\}\right]$ and thus of the same order as terms that we have discarded in our approximation so we neglect it as well. Since the integral over θ forms for values $O(1)$, the constraint on the minimum hopping rate is the same as for the $n = k$ case, i.e., $\omega_{\min}^{-1} \sim \mathcal{O}(T_{\exp})$.

For $n > k$, the maximum $\theta^* \sim (\log\omega_0 t)^{n/k-1}$, which is now large. The dominant order of the derivatives $\partial^p L$ ($p \geq 2$) at $\theta = \theta^*$ is given by $(-1)^{p-1}(p-1)!\,(\theta^*)^{-p-1}$, that is they coincide with those of $\theta^*\log(\theta)$. It follows that $L(\theta) - L(\theta^*) \simeq \theta^*\left[\log(\theta/\theta^*) - (\theta-\theta^*)/\theta^*\right]$. The resulting integral over θ is an Euler Γ-function of (large) argument $\theta^* + 1$: its value is approximated by Stirling formula, which yields $\int (\theta/\theta^*)^{\theta^*} e^{-(\theta-\theta^*)}d\theta \simeq \sqrt{\theta^*}$. The $\sqrt{\theta^*}$ reflects the fact that the integral forms around the maximum at θ^* of the integrand over a range $\sqrt{\theta^*}$, which implies that the approximation $-\log\left(\frac{\theta}{\omega_0 t}\right) \simeq \log(\omega_o t)$ still holds, as in the previous cases $n \leq k$. The $\sqrt{\theta^*}$, as well as the $\log(\omega_0 t)^{1/k-1}$ coming from the denominator in Eq. S3, are subdominant with respect to terms that we have neglected in the expansion of L. We therefore discard them from our final approximation for $n > k$:

$$f(t) \sim t^{-2}\exp\left\{-\frac{a\left(\frac{T_x}{b}\log(\omega_0 t)\right)^{n/k}}{2T_\lambda}\right\}\,.$$

Since the integral over θ forms for values $O\left((\log\omega_0 t)^{n/k-1}\right) \gg 1$, the condition $\omega_{\min}^{-1} \sim \mathcal{O}(T_{\exp})$ ensures *a fortiori* that the finite value of $\omega_{\min}$ does not affect the above result.

Discarding subdominant terms, in all three cases we thus get the general expression we present in the main text,

$$f(t) \sim t^{-2}\exp\left\{-\frac{a\left(\frac{T_x}{b}\log(\omega_0 t)\right)^{n/k}}{2T_\lambda}\right\}\,. \tag{S6}$$

To verify the validity of the above arguments, we show in Fig.S1 how, to dominant order, asymptotic predictions agree with a detailed numerical integration of Eq. S2 for $\Delta U(\lambda) = \lambda^k$ and $V(\lambda) = \lambda^n$.

First-passage time through an absorbing boundary in a harmonic oscillator.—We here derive the first passage distribution for the harmonic oscillator with fixed $s \in \mathbb{R}$, so for

$$dx_t = -(x_t - s x_f)dt + \sqrt{2T_x}dW_t$$

The corresponding Fokker-Planck equation is given by

$$\begin{aligned}\partial_t\rho =& \mathcal{L}\rho\,;\\ \mathcal{L} =& \partial_x((x - s x_f)\bullet) + T_x\partial_x^2(\bullet)\,.\end{aligned}$$

We are interested in the distribution of first passage times from x_0 to the energy barrier located at x_f, derived in the Supplementary Information of [88]. We here reformulate this derivation and tune it to our particular case. The survival probability can be written as

$$S(x_f, t|x_0) = \int_{-\infty}^{x_f} P_{x_f}(x, t|x_0)dx\,,$$

where $P_{x_f}(x,t|x_0)$ is the propagator from $t=0$ to t with the constraint that $x < x_f$. In other words, we have an absorbing boundary condition at x_f, $P(x_f,t|x_0)=0$. From the survival probability, the first passage density can be obtained as

$$f_{x_f}(t|x_0) = -\partial_t S_{x_f}(t|x_0).$$

The backward Kolmogorov equation for the propagator $P_{x_f}(x,t|x_0)$ is $\partial_t P_{x_f}(x,t|x_0) = \mathcal{L}^\dagger_{x_0} P_{x_f}(x,t|x_0)$, where $\mathcal{L}^\dagger_{x_0}$ is the adjoint of the generator of the stochastic process, $\mathcal{L}^\dagger_{x_0} = -(x_0 - sx_f)\partial_{x_0} \bullet + T_x \partial^2_{x_0} \bullet$. Integrating the backward Kolmogorov equation and then taking the time derivative we get

$$\begin{aligned}\partial_t S_{x_f}(t|x_0) &= \mathcal{L}^\dagger_{x_0} S_{x_f}(t|x_0)\\ \partial_t f_{x_f}(t|x_0) &= \mathcal{L}^\dagger_{x_0} f_{x_f}(t|x_0),\end{aligned}$$

which vanishes for $x_0 > x_f$, $f_{x_f}(0|x_0)=0$, $f_{x_f}(t|x_f)=\delta(t)$. To solve for f we take the Laplace transform, $Lf_{x_f}(t|x) = \hat{f}_{x_f}(p|x)$

$$\begin{aligned}\int_0^\infty e^{-pt}\partial_t f_{x_f}(t|x_0)dt =& \mathcal{L}^\dagger_{x_0}\hat{f}_{x_f}(p|x_0)\\ e^{-pt} f_{x_f}(t|x_0)|_0^\infty + p\int_0^\infty e^{-pt} f_{x_f}(t|x_0)dt =& \mathcal{L}^\dagger_{x_0}\hat{f}_{x_f}(p|x_0)\\ f_{x_t}(0|x_0) + p\hat{f}_{x_f}(p|x_0) =& \mathcal{L}^\dagger_{x_0}\hat{f}_{x_f}(p|x_0)\\ (\mathcal{L}^\dagger_{x_0} - p)\hat{f}_{x_f}(p|x_0) =& 0\end{aligned}$$

where we performed integration by parts and made use of the initial condition. The unique solution to the above problem is given by $\hat{f}_{x_f}(p|x_0) = v_p(x_0)/v_p(x_f)$, where $v_p(\bullet)$ is the unique increasing positive solution of the equation $(\mathcal{L}^\dagger_{x_0} - p)v_p = 0$ [59]. For the harmonic oscillator we get

$$-(x - sx_f)\partial_x v_p(x) + T_x \partial_x^2 v_p(x) - p v_p(x) = 0,$$

which we can solve by rewriting $v_p(x)$ as

$$v_p(x) = \exp\left\{\frac{x^2 - 2sx_f x}{4D}\right\} Z_p(x),$$

yielding,

$$T_x \partial_x^2 Z_p(x) + Z_p(x)\left[-p + \frac{1}{2} - \frac{(x - sx_f)^2}{4T_x}\right] = 0$$

Rescaling $x = \sqrt{T_x}y + sx_f$, and using the chain rule, $\partial_x^2 Z_p(x) = T_x^{-1}\partial_y^2 Z_p(y)$, we get,

$$\frac{d^2 Z_p(y)}{dy^2} + Z_p(y)\left(-\frac{y^2}{4} + \frac{1}{2} - p\right) = 0$$

which is Weber's parabolic cylinder differential equation, with solution,

$$Z_p(x) = D_{-p}\left[-\sqrt{T_x^{-1}\left(x - sx_f\right)^2}\right],$$

where D_α is the parabolic cylinder function. Thus,

$$v_p(x) = \exp\left\{\frac{x^2 - 2sx_f x}{4T_x}\right\} D_{-p}\left[-\sqrt{T_x^{-1}\left(x - sx_f\right)^2}\right] \tag{S7}$$

From this we can write an expression for the Laplace transform of the distribution of times for a particle to go from $x_0 = 0$ to $x_f = 1$,

$$\hat{f}_L(p,s) = \hat{f}_{x_f=1}(p|x_0=0) = \frac{v_p(x_0=0)}{v_p(x_f=1)}$$
$$= e^{-\frac{1-2s}{4T_x}} \frac{D_{-p}\left[-\sqrt{s^2/T_x}\right]}{D_{-p}\left[-\sqrt{(1-s)^2/T_x}\right]}, \tag{S8}$$

which we can invert numerically to evaluate the first passage time distribution (see e.g. [89]),

$$f_L(t,s) = L^{-1}\left[\hat{f}_L(p,s)\right]. \tag{S9}$$

In Fig. S2(a) we used the method of de Hoog et al. [60] to numerically invert the Laplace transform and obtain the FPTDs for each fixed s. In addition, we estimate the adiabatic approximation of the full FPTD through,

$$f_L(t) = \int p(s) f_L(t,s) ds, \tag{S10}$$

where $p(s)$ is the distribution of the values of s during first passage time events.

Simulations

Driven harmonic oscillator : We generate 1000 simulations of the Langevin dynamics given in the main text for a slowly-driven harmonic oscillator,

$$\begin{cases} dx_t = -(x_t - s_t x_f)dt + \sqrt{2T_x}dW_t \\ ds_t = -\tau_s^{-1}s_t + \sqrt{2T_s\tau_s^{-1}}dW_t \end{cases},$$

through an Euler-scheme with a sampling time of $\Delta t = 10^{-4}$ s for $T_{\rm exp} = 10^7$ s and with initial condition $x(0) = 0$ and $s(0) \sim \mathcal{N}(0, \sqrt{T_s})$ is sampled according to the Boltzmann distribution. As for the parameter values, we take $T_x = 0.1$, $T_s = 0.1$ Figs. S2, S4, and fix $\tau_s = 10^3 \times T_{\rm exp}$ in the main text and Fig. S3(a).

Driven double well potential: We generate 1000 simulations of Langevin dynamics given in the main text for a slowly-driven double well potential,

$$\begin{cases} dx_t = -4s_t^2 x_t(x_t-1)^2 dt + \sqrt{2T_x}dW_t \\ ds_t = -\tau_s^{-1}(s_t - \mu_s)dt + \sqrt{2T_s\tau_s^{-1}}dW_t \end{cases},$$

through an Euler-scheme with a sampling time of $\Delta t = 10^{-4}$ s for $T_{\rm exp} = 10^7$ s, $\tau_s = 10^3 \times T_{\rm exp}$ and with initial condition $x(0)$ which is randomly chosen as $x(0) = 1$ and $x(0) = -1$ with equal probability and $s(0) \sim \mathcal{N}(\mu_s, \sqrt{T_s})$ is sampled according to the Boltzmann distribution. As for the parameter values, we take $T_x = 0.15$.

Driven parabolic potential: We generate 1000 simulations of the Langevin dynamics given in the main text for a slowly-driven parabolic potential,

$$\begin{cases} dx_t = -\left(x_t - s_t + 2\sin(2\pi k x_t)\right)dt + \sqrt{2T_x}dW_t \\ ds_t = -\tau_s^{-1}s_t + \sqrt{2T_s\tau_s^{-1}}dW_t \end{cases},$$

through an Euler-scheme with a sampling time of $\Delta t = 10^{-4}$ s for $T_{\rm exp} = 10^7$ s, $\tau_s = 10^3 \times T_{\rm exp}$ and with initial condition $x(0) = 0$ and $s(0) \sim \mathcal{N}(0, \sqrt{T_s})$ is sampled according to the Boltzmann distribution. As for the parameter values, we take $T_x = 0.1$.

Numerical integration

We numerically integrate Eq. S2 with $\Delta U(\lambda) = \lambda^k$ and $V(\lambda) = \lambda^n$, through a Riemman sum using the midpoint rule from $\omega_{\min} = 5 \times 10^{-10}$ to $\omega_{\max} = 1$ with $\Delta\omega = 10^{-9}$, yielding the results of Fig. S1.

First passage time distribution estimation

From the simulations of $x(t)$, we first identify all segments, $[t_0, t_f]$, in which t_0 corresponds to the first time x returns to x_0 for the after reaching x_f, and t_f is the time first to reach x_f after t_0. We then build a normalized histogram of first passages times with logarithmic bins. We note that while for the slowly driven potential landscape the position of the minima of the potential are fixed at $x_0 = \pm 1$, for the slowly driven harmonic oscillator and the rugged parabolic potential the minima of the potential are driven by s. For practical reasons, in the numerical estimation of the FPTD we take $x_0 = \langle s \rangle = 0$, which has no practical impact due to the severe separation of time scales between the first passage time events and the relaxation to the well.

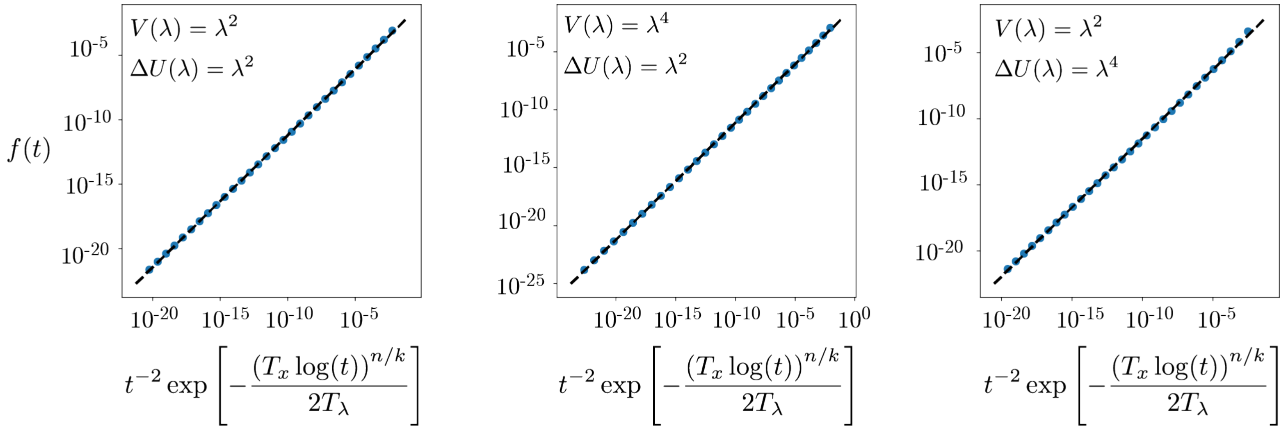

FIG. S1. Numerical integration of $f(t)$ for different choices of $V(\lambda)$ and $\Delta U(\lambda)$, compared to the asymptotic approximation of Eq. S6 (black dashed line) with $T_x = 0.1$ and $T_\lambda = 0.2$.

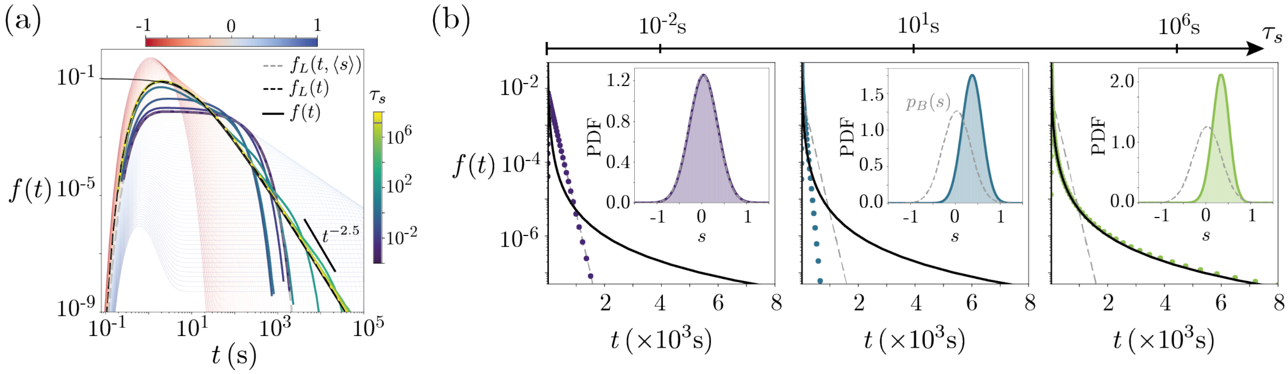

FIG. S2. Emergence of heavy-tails when the driving is sufficiently slow (a) First passage time distribution for different values of τ_s (colored from purple to yellow) show the emergence of heavy tails as $\tau_s \to \infty$. We also plot the full FPTDs $f_L(t, s)$, Eq. (S9), for different fixed values of s (color coded from red to blue). From these estimates, we highlight $f_L(t, \langle s \rangle = 0)$ (gray dashed line), which corresponds to the mean first passage time in the limit $\tau_s \to 0$ (purple line). We also plot $f_L(t)$ (black dashed line), which corresponds to the adiabatic approximation of the FPTD obtained through a weighted average of the FPTDs obtained for fixed s, Eq. (S10); and $f(t)$, which corresponds to Eq. (10) of the main text. (b) First passage times for 3 qualitatively different regimes and the corresponding distribution of s at the first passage event (inset). When $\tau_s \to 0$ (left) the distribution of s at the first passage time events corresponds to the Boltzmann distribution of s, $p(s) \sim e^{-V(s)/T_s}$ (gray dashed line in the inset), and the distribution of first passage times $f(t)$ matches the one obtained from a fixed $s = \langle s \rangle = 0$ (dashed line); when τ_s matches the time scale of x (middle), we observe that the mean first passage time is reduced, boosted by events in which the harmonic potential moves closer to the boundary as shown in the inset; finally, when $\tau_s \gg 1$, we observe the emergence of a heavy tailed first passage time distribution (black line).

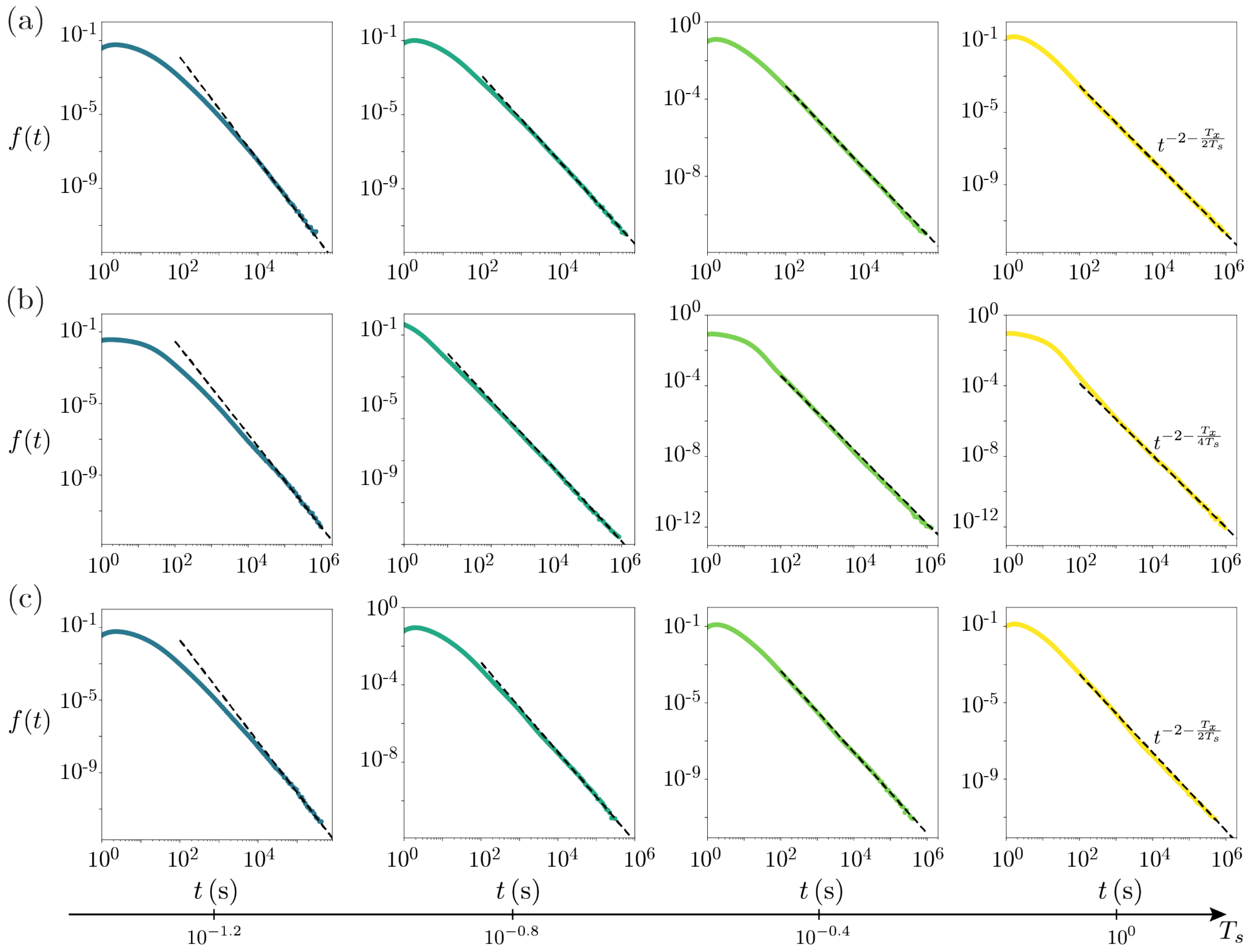

FIG. S3. Details of the accuracy of the asymptotic prediction for the behavior of the tail of $f(t)$ across temperatures T_s for the slowly driven harmonic oscillator (a), the slowly driven double well potential (b) and the slowly driven rugged parabolic potential (c).

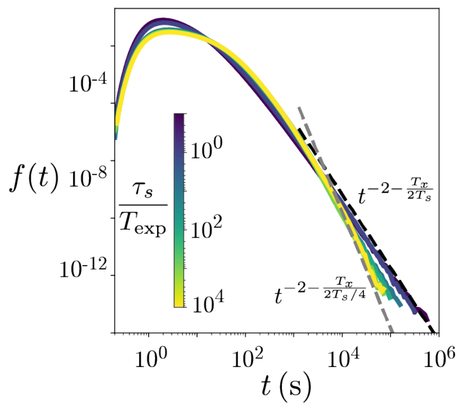

FIG. S4. First passage time distribution for different values of τ_s (colored from purple to yellow) when the initial condition is a narrower Boltzmann distribution with $T_s^0 = T_s/4$. As discussed in the main text, when $\tau_s \to \infty$ the FPTD exhibits a deeper power law exponent. When $\tau_s \sim T_{\rm exp}$ we recover the asymptotic behavior derived in Eq. (10) of the main text.
